# The Chronology of *Angiostrongylus vasorum* (Baillet, 1866), Kamensky, 1905: Infection in *Biomphalaria glabrata* (Say, 1818)

**DOI:** 10.1155/2020/4627158

**Published:** 2020-03-05

**Authors:** T. A. Barçante, J. M. P. Barçante, L. Cardoso, R. N. Remedio, W. S. Lima

**Affiliations:** ^1^Department of Health Sciences, Federal University of Lavras, Lavras, Minas Gerais 37200-000, Brazil; ^2^Department of Parasitology, Federal University of Minas Gerais, Minas Gerais, Brazil; ^3^Department of Veterinary Sciences, School of Agrarian and Veterinary Sciences, University of Trás-os-Montes e Alto Douro (UTAD), 5000-801 Vila Real, Portugal

## Abstract

The intermediate hosts of the French heartworm *Angiostrongylus vasorum* are aquatic and terrestrial gastropods. The present work is aimed at clarifying the sites of penetration and the migratory routes of *A. vasorum* in *Biomphalaria glabrata* snail tissues and evaluating their perilarval reaction with regard to the cellular composition and histological alterations involved in the gastropod response to infection. *Biomphalaria glabrata* snails were individually infected with 1000 first-stage larvae (L1) of *A. vasorum* each and killed at predetermined times after infection. Percutaneous infection occurred simultaneously with oral infection. Despite larval tropism to the fibromuscular tissue, some larvae were located in different tissues and organs. A perilarval reaction was observed around the larvae in a fibromuscular layer, appearing later around the larvae located in the viscera. The number of hemocytes surrounding the larvae increased gradually, forming a pregranuloma. Larval death and degeneration were not observed. No defined migratory pattern occurred, and larval development was apparently not associated with particular tissues or organs. In addition, the infection by *A. vasorum* induces a systemic mobilization of hemocytes in perilarval reaction.

## 1. Introduction

*Angiostrongylus vasorum* (Baillet, 1866) Kamensky, 1905 is a metastrongyloid nematode that parasites wild and domestic canids. Adult worms are found in the right ventricle and pulmonary artery of their hosts, causing cough, dyspnea, decreased exercise tolerance, weight loss, vomit, neurological signs, bleeding disorders, cardiac insufficiency, and ultimately death [1]. The life cycle of *A. vasorum* is heteroxenous, with several aquatic and terrestrial gastropods involved as intermediate hosts, such as snails of the species *Achatina fulica* and *Biomphalaria glabrata* [2–6]. First-stage (L1) larvae are shed in the feces of canids and further develop along two moult to third stage (L3) in the intermediate hosts [7]. The definitive hosts become infected by ingestion of L3, which can be released in the environment or ingested together with a prey (intermediate or paratenic host) [8].

There are several questions concerning the life cycle and the transmission of *A. vasorum* that should be elucidated [4]. Knowledge on the development and larval dynamics in the intermediate hosts are essential to a better understanding of the host-parasite relationship, a circumstance which might contribute to the development of control strategies against angiostrongylosis.

The present work investigated the penetration sites and the chronology of the migratory routes of *A. vasorum* larvae in *Biomphalaria glabrata* and evaluated this intermediate host's perilarval reaction, concerning the cellular composition and the histological alterations involved in the gastropod response to infection.

## 2. Materials and Methods

The *A. vasorum* strain used was isolated from the feces of a naturally infected domestic dog (*Canis familiaris*), living in Caratinga, Minas Gerais, Brazil. The nematodes were maintained by means of serial infections of domestic dogs and aquatic snails (*B. glabrata*) at the Laboratory of Veterinary Helminthology, Department of Parasitology, Federal University of Minas Gerais (UFMG), Brazil. The experimental procedures performed in this work were approved by the UFMG Ethical Committee (protocol number 060/03) and were employed conforming to the accepted principles of animal welfare in experimental science.

### 2.1. Gastropod Infection

First-stage (L1) larvae of *A. vasorum* were obtained from the colony kept in the laboratory. The larvae were recovered from the feces of dogs by the modified Baermann's apparatus [8]. Thirty-eight laboratory-reared *B. glabrata,* ranging from 10 to 15 mm of shell diameter, were individually infected in polystyrene culture test plates with six wells, filled with 3 ml of water and 1000 L1 of *A. vasorum* each. After 24 h, the gastropods were transferred to an aquarium [8].

### 2.2. Chronological Study

During the course of the infection (30 min; 1, 2, 3, 4, 5, 6, 12, and 24 h; and 2, 3, 4, 5, 6, 8, 14, 20, 30, and 60 days), two snails were randomly taken off from the aquarium for assessing each predetermined time of infection. Shells were perforated, and the whole snails were fixed in phosphate-buffered formalin [9], decalcified in 10% EDTA solution for 15 days, for shell demineralization. The soft parts of the gastropods were separated from the shell and placed in an automatic tissue processor for dehydration, diaphanization, embedment, and inclusion in paraffin. The samples were then cut in 5 *μ*m serial sections in a microtome, and one out of six slides was stained with hematoxylin and eosin. The sections were analyzed, and the images were obtained with a bright-field microscope (Carl Zeiss, Germany).

## 3. Results

Both the oral and cutaneous routes were observed for infection. Despite larval tropism to the fibromuscular tissue, some larvae were located in different organs and tissues during the study period, as shown in Table 1. In oral infection, several larvae were observed in the digestive tract lumen. Some of them were located in different layers of the digestive tract wall. Cutaneous infection included the dissociation of the epithelium cells by larvae, which penetrated the tegument and then reached the foot or fibromuscular tissue. However, it is not possible to distinguish the infection route when the larvae were found in the connective tissue, digestive gland, hemocele, or circulatory system. The larvae entering by either of these routes could be located to these tissues.

Most of the larvae were found in the latter, surrounded or not by a cellular aggregate, called here as hemocyte response, with variable degrees of reaction (Table 2).

**Figure 1:**
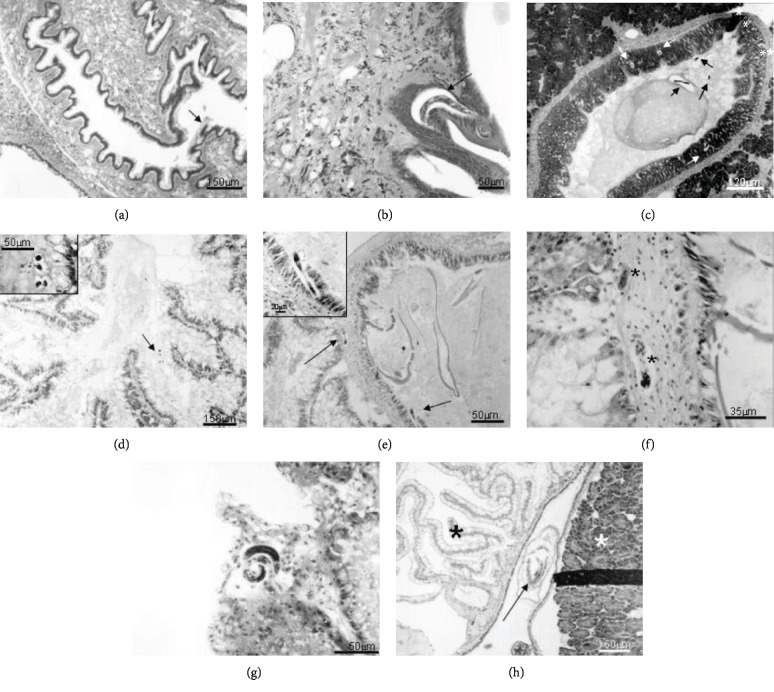
Histological sections of *Biomphalaria glabrata* infected with 1000 L1 of *Angiostrongylus vasorum*. Hematoxylin and eosin staining. (a) Overview of a portion of the digestive tube with a nematode (black arrow) near the mucosa after thirty minutes of the beginning of infection; (b) larva (L1) penetrating in the epithelium of the dorsalis pedis mass of the gastropod after thirty minutes of the beginning of infection; (c) overview of the stomach region with several larvae in the lumen (black arrows), in the epithelium (white arrows), and next to the basal membrane (white asterisks) after one hour of the beginning of the infection; (d) larvae inside of the digestive gland after two hours from the beginning of infection; (e) larva (L1) penetrating the mucosa of the midgut (see expanded detail). In larger arrow, larvae located in the subconjunctival epithelial layer, two hours after the beginning of infection; (f) detail of previous photo showing a discrete local cellular reaction; (g) larva located in the hemocoel of the digestive gland showing a discrete local cellular reaction after three hours of the beginning of infection; (h) larvae inside the circulatory system (arrow) between the albumen gland (white asterisk) and the kidney (black asterisk) after four hours of the beginning of the infection. Magnifications: (a, d) 150 *μ*m; (b, e) 50 *μ*m; (c) 120 *μ*m; (f): 35 *μ*m; (g) 60 *μ*m; (h) 160 *μ*m.

**Figure 2:**
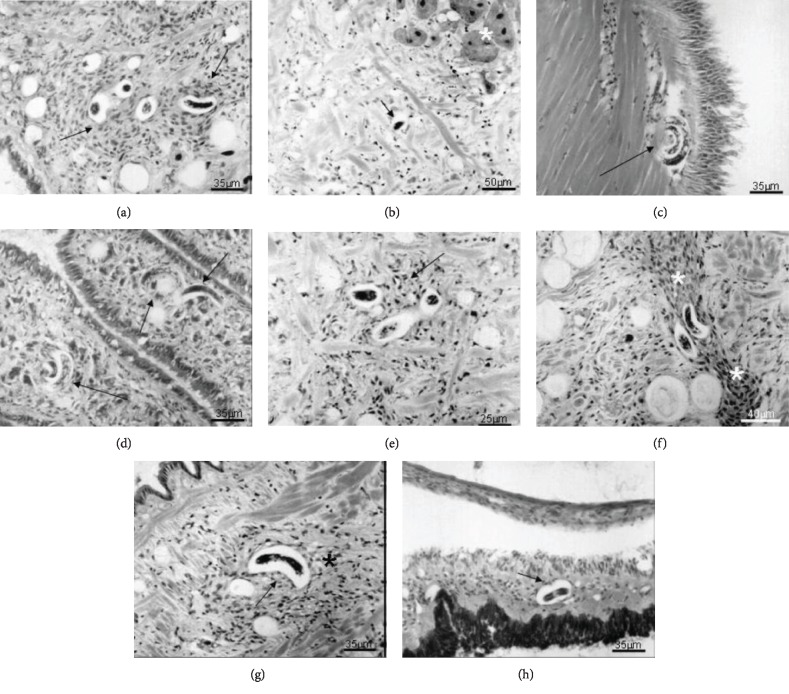
Histological sections of *Biomphalaria glabrata* infected with 1000 L1 of *Angiostrongylus vasorum*. Hematoxylin and eosin staining. (a) Larva in the foot fibromuscular tissue (arrows) presenting a clear space between it and the tissue with cellular infiltrate around five hours after the beginning of infection; (b) larvae in foot fibromuscular tissue with no evident cellular reaction with six hours after beginning of infection. White asterisk shows structures of the nervous system; (c) larva located in the columellar muscle with cellular infiltrate close to it; (d) larvae located in the lamellae of pseudobranquia (arrow), showing no cellular reaction, eight hours after the beginning of infection; (e) larvae in foot fibromuscular tissue (arrow) with evidence of perilarvar reaction more organized, maintaining a clear space of twelve hours after the beginning of infection; (f) intense cellular reaction in the fibromuscular tissue of dorsalis pedis mass with the presence of a pregranuloma perilarvar (white asterisk) twenty-four hours of the beginning of infection; (g) cell infiltration in the fibromuscular tissue of the dorsalis pedis region (black asterisk) with the starting to form a cell button on the one side of the pregranulomatous reaction (arrow); (h) larva located in the mantle without showing obvious cellular reaction maintaining the standard of formation of a clear space with forty-eight of the beginning of infection. Magnifications: (a, c, d, g, and h) 35 *μ*m; (b) 50 *μ*m; (e) 25 *μ*m; (f) 40 *μ*m.

At 30 min of infection (Figures 1(a) and 1(b)), the presence of a greater number of L1 in the esophagus and anterior and posterior intestine was detected. From the first hour (Figure 1(c)), the larvae were observed adhering to the mucosa of the stomach and in an intraepithelial location. In both cases, no cellular reaction was observed. With 2 h of exposure (Figures 1(d)–1(f)), they were observed in the digestive gland and inside the diverticula, with no perilarvar cell response. In addition, the larvae (L1) were also observed in the subepithelial conjunctival layer between the midgut and the digestive gland, with a slight local cellular reaction. After 3 h of infection (Figure 1(g)), the larvae (L1) adjacent to the digestive gland and free in the hemocele, with mild cellular reaction, were visualized; in the stomach, the larvae were observed in an intraepithelial location or passing through the basal membrane, with a discrete cell infiltrate. At 4 h of infection (Figure 1(h)), L1 were first observed in the circulatory system and a larva in the mantle cavity. Larvae present in the pedal region and involved by pregranuloma were observed after 5 h of infection (Figure 2(a)). After 6 h (Figures 2(b) and 2(c)), some parasites were visualized in the fibromuscular tissue, still without perilarvar reaction; in the tissue adjacent to the buccal bulb, a local cell reaction could be observed, although the presence of a larva within the columellar muscle promoted cell reaction. At 8 h of infection (Figure 2(d)), some larvae were found in the foot tissue and pseudogill; at 12 h (Figure 2(e)), these parasites were observed for the first time in the lining epithelium of the pulmonary cavity and pulmonary diverticula.

**Figure 3:**
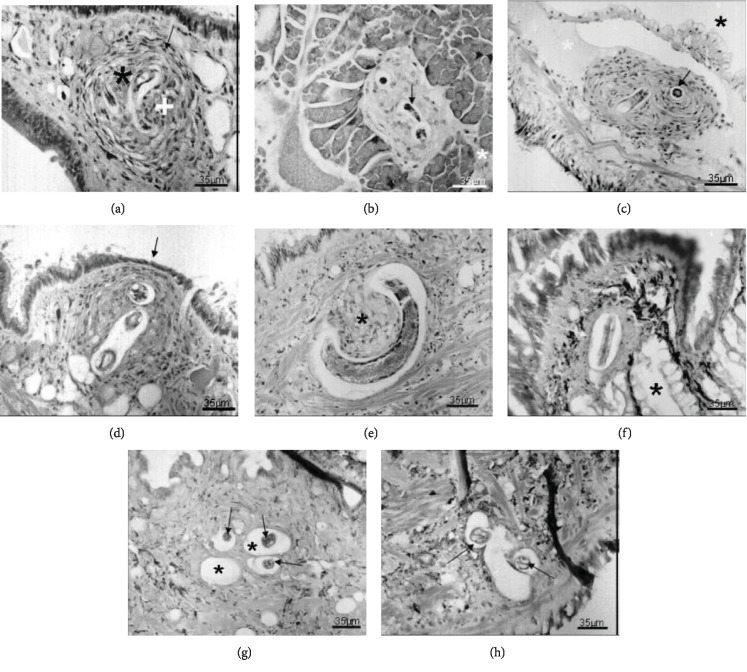
Histological sections of *Biomphalaria glabrata* infected with 1000 L1 of *A. vasorum*. Hematoxylin and eosin staining. (a) Granuloma formation of two distinct layers, an internal with cells of epithelioid aspect (black asterisk) and outer, with cells like fibroblast (arrow). The white cross shows the formation of the cell button on the one side of the larva after six days of the beginning of infection; (b) detail of the larva (arrow) within the albumen gland being surrounded by a cell reaction; the white asterisk shows the cell reaction present six days after the beginning of infection; (c) granuloma formation within the renal artery (white asterisk) fourteen days after the beginning of infection. Renal space is represented by the black asterisk, and the larva indicated by the arrow is an L2 because this has granules around the intestine (14 days); (d) granuloma in the dorsalis pedis region determining a thinning of the epithelium (arrow) fourteen days after the beginning of infection; (e) larva in the foot fibromuscular tissue with a strong reaction in the form of button (black asterisk) without typical presentation of granuloma around, after twenty days of the beginning of infection; (f) larva (white arrow) in a less typical granuloma located in the conjunctive tissue around the kidney (black asterisk) thirty days after the beginning of infection. (g) Weak cellular reaction around the larva with looser appearance. It can be observed larvae within mucus cells (black asterisk) thirty days from the beginning of infection; (h) larvae located in foot fibromuscular tissue without presenting typical granulomatous response, keeping the space even more clear after thirty days at the beginning of infection. Magnifications: (a–h) 35 *μ*m.

**Figure 4:**
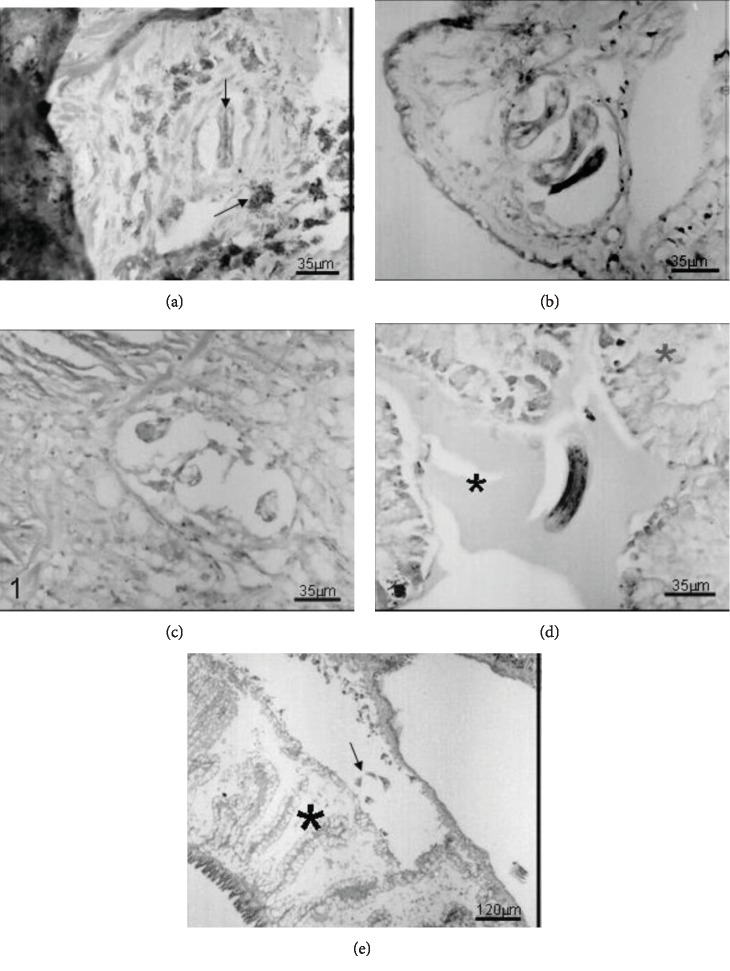
Histological sections of *Biomphalaria glabrata* infected with 1000 L1 of *A. vasorum*. Hematoxylin and eosin staining. (a) Larva (vertical arrow) located in the foot fibromuscular tissue with loss of cellularity and loose appearance of the tissue plus deposit of melanin pigment (oblique arrow) after sixty days of the beginning of infection; (b) larvae (L3) in pedal tissue without evidence of granulomatous structure showing a tissue with very loose aspect sixty days after the beginning of infection; (c) larvae (L3) in conjunctive tissue with loose appearance and presence of well-marked clear space around the larvae sixty days after the beginning of infection; (d) free larva (L3) located in spaces (black asterisk) between the diverticula of the digestive gland (gray asterisk) without the presence of cellularity around after sixty days of the beginning of infection; (e) free larva (arrow) in the renal space (black asterisk) without the presence of cellularity sixty days after the beginning of infection. Magnifications: (a–d): 35 *μ*m; (e): 120 *μ*m.

After 1 day (Figures 2(f) and 2(g)), most of the parasites were located in the muscular tissue of the pedal mass, surrounded by a more intense cell reaction with pregranuloma formation, in some cases showing cell migration to the concave surface of the larvae. The larvae (L1) were observed in the mantle cavity epithelium and lung tissue after 48 h (Figure 2(h)). After 6 days (Figures 3(a) and 3(b)), several typical granulomas were distributed in the pedal region and, for the first time, the parasites were found housed in the interstitial of the albumen gland. The larvae were also found in the renal circulatory system and hemocele, the connective tissue, between the diverticula of the digestive gland and pseudogill of infected snails after 14 days (Figures 3(c) and 3(d)). At 20 days of infection (Figure 3(e)), the observed pattern was similar, although with different tissue reactions. At 30 days (Figures 3(f)–3(h)), the larvae were located in the pedal mass, kidney, pseudogill, right-rectal tissue, and the conjunctive tissue near the columellar muscle, and at 60 days of infection (Figures 4(a)–4(e)), they were found free in the hemolymph and in the pedal mass and in the hemocele, with a very weak reaction.

The first and discreet sign of hemocyte response appeared at 2 h in the midgut, when a single layer of hemocytes surrounded some of the larvae (L1) (Figures 1(e) and 1(f)). The number of hemocytes surrounding the larvae gradually increased, and from 5 h after infection, these cells were more organized, forming a pregranuloma (Figures 2(a)–2(f)). The hemocyte response varied, and different larvae presented responses with different intensities, or even the absence of a response. A clear space around the larvae was present in all specimens analyzed (Figures 2(e)–2(h)). At 24 h post infection, the response was more intense and frequently formed pregranulomas and an aggregate of hemocytes in the concavity of the larvae, called cell button (Figure 2(g)). The formation of granulomas was observed at day 6 of infection. Granulomas presented two layers, an internal thick layer formed by several hemocytes concentrically organized and an external layer, of flattened hemocytes, forming a capsule (Figures 3(a) and 3(b)). Cytoplasmic vacuoles indicating the high activity of the hemocytes composing the granuloma were seen at day 14 (Figures 3(c) and 3(d)). Occasionally, the epithelium was stretched, due to the pressure generated by the growing granulomas. The extension of the hemocyte reaction and the amount of hemocytes apparently decreased at 20 days of infection (Figure 3(e)). Larvae (L2) with and without a surrounding hemocyte response were located close to the subepithelial tissue of the rectum. After 30 days, granulomas did not present the external layer of flattened cells and the aggregate of hemocytes was looser, smaller, with a spongy appearance, and sometimes presenting melanin pigments (Figures 3(f)–3(h)). The appearance of the granulomas throughout the organs changed at 60 days. They became looser and like spongy tissue in appearance. The clear space around the larvae was wider, and the hemocyte infiltrate was almost absent (Figures 4(a)–4(e)).

## 4. Discussion

Since 1960, when gastropods of the genus *Arion* were proved to participate in the life cycle of *A. vasorum* [7], a few new investigations focused on the host-parasite relationship were reported [4, 8]. The present study has focused on the histological aspects of *B. glabrata* infection by *A. vasorum*. It was possible to observe oral and cutaneous infection, followed by L1 spread to several tissues and organs, and the modification of the hemocyte response surrounding the larvae.

Nematodes of the order Metastrongyloidea, including *A. vasorum*, infect their intermediate hosts penetrating their digestive tract and/or tegument [10–15]. In the present work, larval invasion through the digestive tract wall occurred in successive steps, showing no preference to specific segments, as previously observed by other authors [16–18]: the adhesion to the epithelium surface, the transepithelial migration to the basal membrane, the transposition of the basal membrane, and the invasion of the connective subepithelial tissue. The evidences of cutaneous infection included the observation of larvae penetrating the epithelia and the presence of L1 right above the tegument in the first minutes and hours of infection. These two infection pathways have also been confirmed in other angiostrongylids [10, 11, 13, 15]. In fact, infection of *Angiostrongylus costaricensis* occurs concomitantly also through oral and percutaneous pathways, as observed in histological sections [19]. However, the molecular mechanisms that participate in the interaction between L1 larvae and the epithelium from different segments of the digestive tract of gastropods are still unknown.

In the present study, the possible decrease in the number of L3 inside the specimens with 30 and 60 days of infection may indicate that larval elimination occurred. During this period, larval morphology did not evidence abnormalities, showing that the larvae survived inside their hosts. These facts indicate that the larvae are being eliminated in the environment, as suggested by other authors [20]. Conboy et al. [21] reported the detection of spontaneously shed metastrongyloid L3 in the feces of experimentally infected slugs. In the present study, the presence of L3 larvae close to the rectum may also suggest that this region probably acts as a route for larval elimination of *A. vasorum.*

During the process of infection and migration within the hosts, the larvae may be retained in some places, mainly in the fibromuscular tissue, surrounded or not by hemocyte reaction. However, some larvae (L1) spread through the organism, moving in the tissues with the help of enzymes such as proteases or enter the circulatory system and flow in vessels and sinuses until they reach a tissue or organ. In the present work, penetration and invasion occurred preferentially in the first 5 h. However, migration through viscera occurs for a longer period of time. Larvae preferably reach the digestive gland, kidney, gastrointestinal tract, lung, pseudobranchia and connective tissue.

Several works have also found a large amount of *Angiostrongylus* larvae in the fibromuscular tissue [14, 16, 17]. Montresor et al. [17] suggests that a great number of larvae of *A. costaricensis* in the fibromuscular tissue results from the ample hemolymphatic vascularization in this tissue. Richards and Merritt [14] proposed that the majority of *A. cantonensis* larvae that penetrates through the digestive tract wall reach the hemocoel, leading to the fibromuscular tissue and foot.

The development of perilarval reaction started from 5 h of infection and showed a development pattern independent from the destination of the larvae. However, the larvae (L1) penetrating the integument may attach to the foot fibromuscular tissue, whereas those which were ingested needed to cross the intestinal epithelium at any level and without specificity to the site of penetration, in order to reach the other internal organs through hemocoel or by direct contact. In addition, the larvae may reach the various organs apparently at any time, since they are ingested at the beginning of the exposure time.

Cellular response in gastropods is performed by hemocytes (coelomocytes or amoebocytes), a phagocyte cell that cluster (encapsulate) around pathogens and produce humoral intermediates. In the present study, we observed that the larvae were not damaged by the hemocyte reaction and the tissues and organs of the snails were not degenerated. Hemocyte reaction was concentrated around the larvae, and except for these points of hemocyte aggregation, the tissues and organs presented a normal appearance. The parasite was able to develop well, even when surrounded by hemocytes. For example, metastrongyloid larvae are known to survive inside the cellular reaction [16, 22]. Some studies also discuss the host's immune response exploitation by parasites [23, 24], and the encapsulation of angiostrongylids has been considered an example of such a process [16, 17, 22].

Initially, a cell density was established around the larvae, with subsequent differentiation of fibroblast-like cells, forming a pseudocapsule from 6 days of infection. Other authors [25, 26] also proposed this differentiation of amoebocytes in fibroblasts. This same pattern was also observed in *A. cantonensi*s infections in *B. glabrata*. The formation of a clear space around the larvae is a common phenomenon in other angiostrongylids, and it is attributed to potential proteolytic enzymes produced by the larvae [16]. However, this histological pattern should not be confused with those larvae that enter mucus cells in the foot, which have similar appearance, and also with the possibility of the occurrence of tissue retraction, a histological artifact. In the present study, it was not possible to prove that larval proteolytic activity is responsible for the clear spaces separating larval sections and surrounding host tissue. However, the clear spaces were observed in several samples. This suggests that it is necessary to investigate if this phenomenon is due to proteolytic enzymes as already proved to *A. costaricensis* infecting *Sarasinula marginata* [16].

This work showed that infection of *B. glabrata* by *A. vasorum* occurs by oral and percutaneous pathways, without a defined migratory pattern. Likewise, in the site of infection, a systemic mobilization of hemocytes is induced, causing the perilarval reaction, although without the death of larvae. The growing veterinary importance of the nematode *A. vasorum* and the issues about its zoonotic potential reinforce the importance of studies on the host-parasite relationship. Studies comparing the morphological aspects of the hemocyte reaction to *A. vasorum* infection in different species of gastropods could contribute to a better understanding of the innate immune system in invertebrates. Besides, they may also be used as tools for the study and development of control methods for this parasite.

## Figures and Tables

**Table 1:** Larvae of *Angiostrongylus vasorum* situated in different tissues and/or organs after infection of *Biomphalaria glabrata.*

Infection time	Larvae location																	
	PE	FB	CS	Hm	DT	AM	CE	CM	CT	DG	MC	BB	CM	PS	ME	AG	CT	KD
30 min	+	-	-	-	+	+	-	-	-	-	-	-	-	-	-	-	-	-
1 h	-	-	-	-	+	+	+	+	-	-	-	-	-	-	-	-	-	-
2 h	-	-	-	-	+	-	+	-	+	+	+	-	-	-	+	-	-	-
3 h	-	-	-	-	+	-	+	+	+	+	+	-	-	-	-	-	-	-
4 h	-	-	+	-	+	-	-	-	-	-	+	-	-	-	-	-	-	-
5 h	-	+	-	-	+	-	-	-	-	-	-	-	-	-	-	-	-	-
6 h	-	+	-	-	+	+	-	-	-	-	-	+	+	-	-	+	-	-
8 h	-	+	-	-	-	+	-	-	-	-	-	-	-	-	-	-	-	-
12 h	-	+	-	-	+	-	-	-	-	-	-	-	-	-	-	-	-	-
24 h	-	+	-	-	+	-	-	-	-	+	-	-	-	-	-	-	-	-
2 days	-	+	-	-	-	-	-	-	-	-	-	-	-	-	-	-	-	-
3 days	-	+	-	-	-	-	-	-	-	-	-	-	-	-	-	-	-	-
4 days	-	+	-	-	-	-	-	-	-	-	-	-	-	-	-	-	-	-
5 days	-	+	-	-	-	-	-	-	-	-	-	-	-	-	-	-	-	-
6 days	-	+	-	-	-	-	-	-	-	-	-	-	-	-	-	-	-	-
8 days	-	+	-	-	-	-	-	-	-	-	-	-	-	+	-	-	-	-
14 days	-	+	-	+	-		-	-	-	+	-	-	-	+	+	-	+	+
20 days	-	+	-	+	-	-	-	-	-		-	+	-	+	+	-	+	+
30 days	-	+	-	+	-	-	-	-	-	-	-	-	-	+	-	-	+	+
60 days	-	+	+	+	-	-	-	-	-	-	-	-	-	-	-	-	+	+

PE: penetrating epithelia; FB: fibromuscular tissue or foot; CS: circulatory system (vessels or sinus); Hm: hemocele; DT: lumen of digestive tract; AM: adjacent to the mucosae; CE: crossing the epithelia; CM: crossing basement membrane; CT: connective tissue; DG: digestive gland; MC: mantle cavity; BB: buccal bulb; CM: columellar muscle; PS: pseudobranquia; ME: mantle epithelia; AG: albumen gland; CT: connective tissue between organs; KD: kidney.

**Table 2:** Morphological classification of the hemocyte response of *Biomphalaria glabrata* infected by *Angiostrongylus vasorum.*

Infection time	Classification of hemocyte response						
	Absent	Discrete	Moderate	Pregranuloma	Granuloma	Aggregate in the concavity of the larvae	Loose aggregate hemocytes
30 min	X						
1 h	X						
2 h		X					
3 h		X					
4 h		X					
5 h			X	X			
6 h			X	X			
8 h			X	X			
12 h			X	X			
24 h			X	X		X	
2 days				X			
3 days							
4 days							
5 days							
6 days						X	
8 days							
14 days					X		
20 days				X	X	X	
30 days							X
60 days							X

## Data Availability

The data used to support the findings of this study are included within the article.
